# Influences of Strain Rates on Tensile and Shear Performances of CF/PP and GF/PP Thermoplastic Composites

**DOI:** 10.3390/polym17182446

**Published:** 2025-09-10

**Authors:** Changye Liu, Juncheng Lv, Yixin Chen, Xinyue Miao, Qinghao Liu, Zhen Wang, Guohua Zhu, Kai Song

**Affiliations:** 1 SAIC-GM-Wuling Automobile Co., Ltd., Liuzhou 545007, China; 2 School of Automation and Intelligent Manufacturing, Southern University of Science and Technology, Shenzhen 518005, China; 3 School of Automobile, Chang’an University, Xi’an 710064, China; 4 State Key Laboratory of Advanced Design and Manufacturing Technology for Vehicle, Hunan University, Changsha 410082, China

**Keywords:** thermoplastic composite, mechanical characterization, strain rate dependency, failure mechanism, analytical model

## Abstract

This study aims to investigate the influences of strain rates on tensile and shear performances of carbon fiber-reinforced polypropylene (CF/PP) and glass fiber-reinforced polypropylene (GF/PP) thermoplastics. First, the differential scanning calorimetry (DSC) and thermogravimetric analysis (TGA) tests were conducted on the polypropylene to determine its melting and decomposition temperatures, identified as approximately 166 °C and 450 °C, respectively. Subsequently, CF/PP and GF/PP specimens were fabricated through the thermo-compression molding process, and subjected to the uniaxial tension and bias extension tests across six strain rates (1.7 × 10^−6^ s^−1^, 0.5 s^−1^, 5 s^−1^, 50 s^−1^, 250 s^−1^, and 500 s^−1^). The results indicated that the tensile modulus/strength and shear modulus/strength of both CF/PP and GF/PP specimens improved with the increase in strain rates, whereas the shear failure strain exhibited a decreasing trend due to the transition of polypropylene from ductile to brittle behaviors. At 500 s^−1^, CF/PP exhibited 53.08%/53.6% and 52.5%/52.4% increases in tensile/shear modulus and tensile/shear strength compared to 1.7 × 10^−6^ s^−1^, while GF/PP showed 54.6%/113.4% and 71.5%/92.3% improvements, respectively. Furthermore, fracture surfaces exhibited progressive roughening with increasing strain rates. The dynamic increase factor (DIF) quantitatively characterized the strain rate dependencies of elastic and strength properties, establishing an analytical model for developing rate-dependent constitutive models in future research.

## 1. Introduction

Automotive industries face escalating challenges in vehicle lightweight development, including achieving further weight reduction, balancing cost with performance, adopting efficient and environmentally friendly production methods, and enhancing protection capabilities [1,2,3,4]. Thermoplastic composites, as a prominent class of advanced lightweight materials, are renowned for their recyclability, good toughness, undemanding storage requirements, excellent formability, and low density [5,6,7,8]. These attributes make them a promising engineering material for widespread adoption in modern automobiles. However, despite these significant advantages, the broader application of thermoplastic composites is hindered by their complex mechanical behaviors, which exhibit inherent inelasticity [9,10,11], strong rate dependence [12,13,14], and significant temperature dependence [15,16,17] characteristics. Consequently, confronted with these sophisticated technical hurdles and intense international market competition, automotive manufacturers must drive continuous innovation to address these mechanical challenges and thereby meet evolving customer demands for enhanced performance and safety.

Initially, thermoplastic materials were primarily employed in vehicles for interior and exterior trim parts, door panels, and underbody closures. Subsequently, as material properties improved and manufacturing technologies advanced, researchers increasingly focused on the crashworthiness of thermoplastic composite energy-absorbing structures [18,19]. This led to the gradual emergence of thermoplastic crashworthy components such as front bumpers [20], B-pillars [21], and dedicated energy-absorbing devices [22]. During an actual collision, crashworthy structures within a vehicle body can experience diverse deformation patterns under varying strain rates. Therefore, designing effective energy-absorbing structures requires a fundamental understanding of dynamic mechanical responses of thermoplastic composites, especially necessitating characterization of their rate-dependent characteristics through experimental tests. Integrating these physical data with numerical analysis establishes a virtual design framework that can significantly accelerate development cycles and reduce costs for automakers.

Extensive studies have characterized dynamic tensile responses of thermoplastic polymers, including polypropylene (PP) [23,24,25], polyamide (PA) [26], and Polyetheretherketone (PEEK) [27], confirming their strain rate dependence. Additionally, prior research has also investigated the tensile behavior of both short and long fiber-reinforced thermoplastic composites under varying strain rates [28]. Notta-Cuvier et al. [29] explored the effects of both strain rates and fiber orientations on the mechanical properties of short glass-reinforced plastics at micro/macroscopic scales, and they demonstrated strain rate sensitivity in both net PP and PP-30GF composites. Chen et al. [30] analyzed the tensile behaviors of short carbon/glass fiber-reinforced PEEK composites with a strain rate ranging from 10^−3^ s^−1^ to 10^3^ s^−1^, and reported strain rate insensitivity in tensile strength for both PEEK matrix and its composites, while the failure strains exhibited rate-dependent trends. Abdo et al. [31] observed significant rate-dependent features in tensile strength of short glass fiber-reinforced PBT composites over strain rates of 0.003 s^−1^ to 0.6 s^−1^. Lienhard et al. [32] studied the dynamic tensile and shear behaviors of long glass fiber-reinforced PP composites, observing increased energy absorption capacity under dynamic loading compared to static conditions (from 10^−3^ s^−1^ to 10^2^ s^−1^).

Compared with short/long fiber-reinforced composites, continuous fiber reinforcements exhibit superior mechanical properties. Woven fabric-reinforced composites are especially well suited for automotive crashworthy structures owing to their balanced in-plane mechanical performances. Coussa et al. [33,34] demonstrated that the shear responses of glass fabric-reinforced PA66 thermoplastics exhibit a pronounced sensitivity to variations in strain rate (5 × 10^−3^ s^−1^~25 s^−1^). Todo et al. [35] experimentally studied the tensile responses of both carbon and glass fabric-reinforced PA6/modified PA6 (mPA6) thermoplastics across strain rates ranging from 0.01 s^−1^ to 40 s^−1^, and found that mechanical properties of composites containing PA6 exhibit greater sensitivity to strain rate variations compared to those containing modified mPA6. Brown et al. [36] studied the dynamic tensile, in-plane shear and compressive responses of glass fabric-reinforced PP thermoplastics, and found increased tensile/compressive stiffness and strength but decreased shear properties with rising strain rates in glass/PP composites (1.7 × 10^−6^ s^−1^~70 s^−1^). Despite stiffness/strength disparities between thermoplastic and thermoset composites, PEEK-/PPS-based thermoplastics can achieve comparable performances [37,38,39,40]. However, the high material cost of PEEK and PPS limits their widespread adoption in automotive applications. In contrast, low-cost and widely sourced PA and PP matrix offers a cost-effective alternative.

The existing literature predominantly concentrates on the short/long fiber-reinforced PA and PP thermoplastics, with limited research addressing the dynamic mechanical properties of woven carbon and glass fabric-reinforced PA and PP thermoplastics. Although some prior studies have investigated the strain rate effects in carbon or glass fabric-reinforced PA and PP thermoplastic composites [33,34,35,36], considerable knowledge gaps still remain. Notably, there is a scarcity of data concerning woven carbon and glass fabric-reinforced thermoplastics subjected to strain rates exceeding 250 s^−1^, particularly regarding shear properties. Furthermore, no comprehensive model currently exists that quantifies the rate dependency across both tensile and shear modes for woven carbon and glass fabric-reinforced thermoplastics. This limitation hinders the development of rate-dependent constitutive models necessary for dynamic impact numerical simulations. This work aims to address these gaps by characterizing tensile/shear behaviors of both woven carbon fiber and glass fiber-reinforced PP thermoplastics across six strain rates (1.7 × 10^−6^ s^−1^~500 s^−1^), covering previously unreported high-rate regimes, and proposing a dynamic increase factor (DIF) model with material-specific parameters for supporting the development of rate-dependent constitutive models.

This study experimentally investigated both tensile and shear behaviors of woven carbon fiber-reinforced polypropylene (CF/PP) and woven glass fiber-reinforced polypropylene (GF/PP) thermoplastic composites, accounting for six strain rates (1.7 × 10^−6^ s^−1^, 0.5 s^−1^, 5 s^−1^, 50 s^−1^, 250 s^−1^, and 500 s^−1^). First, differential scanning calorimetry (DSC) and thermogravimetric analysis (TGA) characterized the melting and decomposition temperatures of PP matrix to determine the thermo-compression molding parameters. Subsequently, both CF/PP and GF/PP laminates were fabricated and subjected to uniaxial tension and bias extension tests at prescribed strain rates. Finally, strain rate dependencies in deformation modes and mechanical properties of CF/PP and GF/PP composites were quantitatively analyzed.

## 2. Materials and Tests

### 2.1. Thermal Properties of Polypropylene

Differential scanning calorimetry (DSC) and thermogravimetric analysis (TGA) were employed to characterize crucial thermal properties of the PP matrix, specifically determining its melting and decomposition temperatures. The DSC and TGA testing equipment was provided by Netzsch (BAV, Germany). For DSC testing, PP samples underwent heating from 25 °C to 250 °C at 5 °C /min; concurrently, TGA testing followed identical heating rates over a 30 °C to 550 °C range. Both methodologies implemented nitrogen atmosphere protection to prevent oxidative degradation. The crystallinity ($X_{C}$) was subsequently derived from DSC thermograms using the following calculation [41]:

(1) $$X_{C}=\frac{H_{m}}{(1-M_{f})\times H_{m}^{0}}\times 100\%$$

where $H_{m}$ denotes the melting temperature, $H_{m}^{0}$ is the melting enthalpy, and $M_{f}$ represents the weight fraction of the fiber reinforcements.

**Figure 1:**
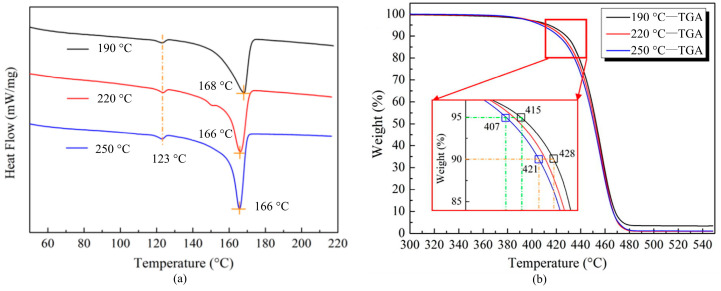
Characterizations of thermal properties of polypropylene: (**a**) heat flow–temperature curves of DSC tests; (**b**) weight–temperature curves of TGA tests.

To ensure the credibility of the results, three different samples were fabricated through the thermo-compression molding technology with three different temperatures (190 °C, 220 °C, 250 °C). Figure 1a,b illustrate the heat flow/temperature curves obtained from the DSC tests and weight/temperature curves from the TGA tests, respectively. It can be found that the ultimate melting temperature of the PP matrix was about 166 °C, as shown in Figure 1a. Meanwhile, the thermogravimetric data in Figure 1b indicate thermal decomposition onset near 400 °C, and the decomposition temperature of the PP matrix ranged between 450 °C and 480 °C, in which the sample lost the vast majority of weight.

### 2.2. Preparation of Specimens

**Figure 2:**
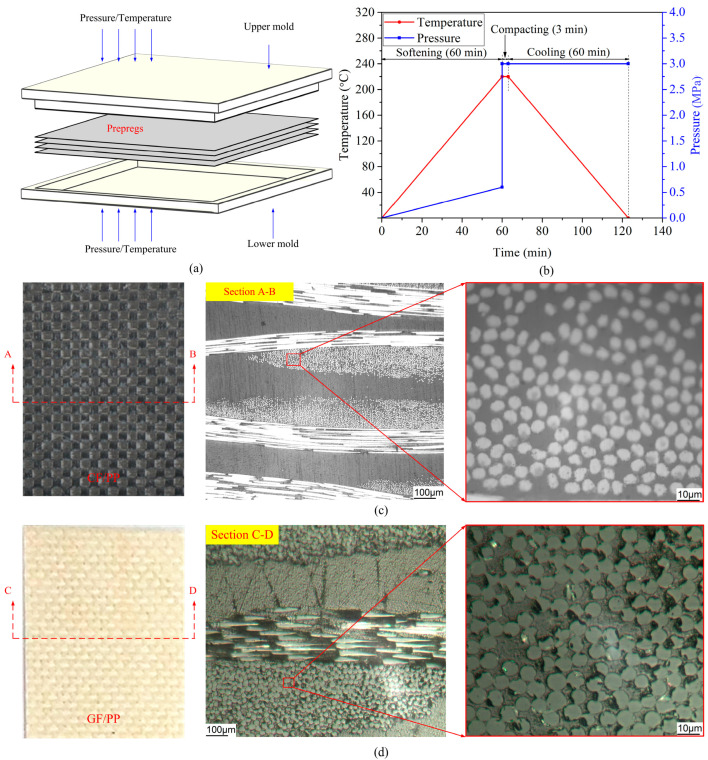
Thermo-compression molding process: (**a**) schematic diagram of the thermo-compression molds; (**b**) process curves; (**c**) macro-/micromorphologies of CF/PP; (**d**) macro-/micromorphologies of GF/PP.

Figure 2a,b present a schematic diagram of the thermo-compression molds and the corresponding process curve, respectively. The thermo-compression molding process was conducted utilizing a hot press machine (HBSCR-300T/600-900A, provided by Qingdao Huabo, Qingdao, China). The laminate fabrication process includes sequential stages: ply stacking, preheating, compaction, demolding, and trimming. The consolidated six-ply CF/PP and GF/PP laminate exhibited average thicknesses of 2.1 mm and 2.2 mm, respectively. Figure 2c,d illustrate the representative macro-/micromorphologies of the CF/PP and GF/PP specimens, respectively. Table 1 presents a summary of the material compositions and dimensional specifications of the CF/PP and GF/PP prepregs.

### 2.3. Dynamic Tensile Tests

**Figure 3:**
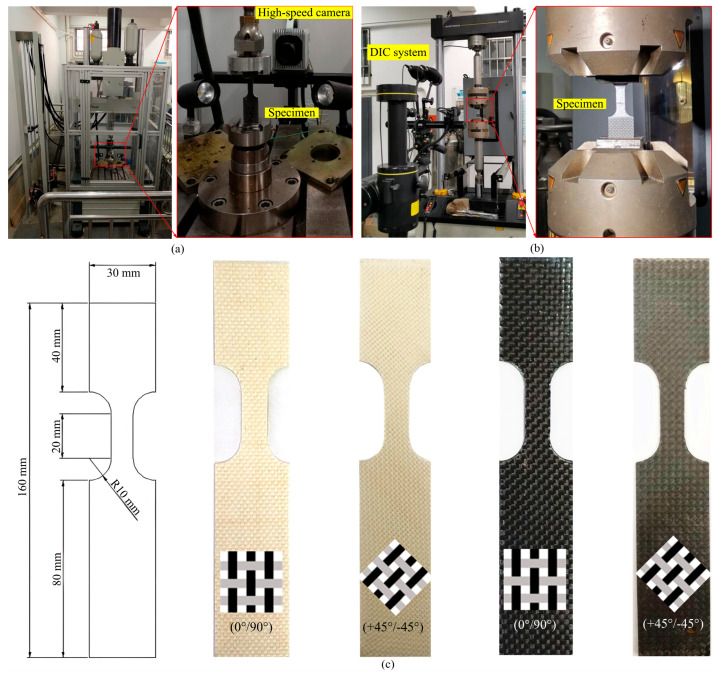
Equipment and specimens: (**a**) high-speed tensile machine (0.01 m/s~20 m/s); (**b**) quasi-static tensile machine (2 mm/min); (**c**) geometric dimensions of testing specimens.

To capture deformation modes and strain evolution during dynamic tensile tests, a high-speed camera integrated with digital image correlation (DIC) was employed for non-contact strain measurement. All dynamic tests were conducted using the high-speed tensile machine (HTM 5020, ZwickRoell, Ulm, Germany), which offers a velocity range from 0.01 m/s to 20 m/s, as illustrated in Figure 3a. Since the lowest velocity of the machine is limited to 0.01 m/s, quasi-static tests with velocity of 2 mm/min (strain rate of 1.7 × 10^−6^ s^−1^) were conducted at the static tensile machine (8801, Instron, Norwood, MA, USA), and the DIC system was also employed to capture the strain evolution and failure behaviors of specimens, as shown in Figure 3b. Figure 3c shows the CF/PP and GF/PP specimens subjected to uniaxial tension and bias extension tests.

Uniaxial tension and bias extension tests were performed on CF/PP and GF/PP specimens in accordance with ASTM D3039 and ASTM D3518 standards [36], respectively. Six strain rates were investigated in this study, including 1.7 × 10^−6^ s^−1^ (3.3 × 10^−5^ m/s), 0.5 s^−1^ (0.01 m/s), 5 s^−1^ (0.1 m/s), 50 s^−1^ (1 m/s), 250 s^−1^ (5 m/s), and 500 s^−1^ (10 m/s). Each experimental condition was replicated three times, with the mean values reported as the definitive results. Fiber orientations of (0°/90°) and (+45°/−45°) were employed for uniaxial tension and bias extension tests, respectively. Specimen details are summarized in Table 2.

## 3. Results and Discussion

### 3.1. Mechanical Behaviors

**Figure 4:**
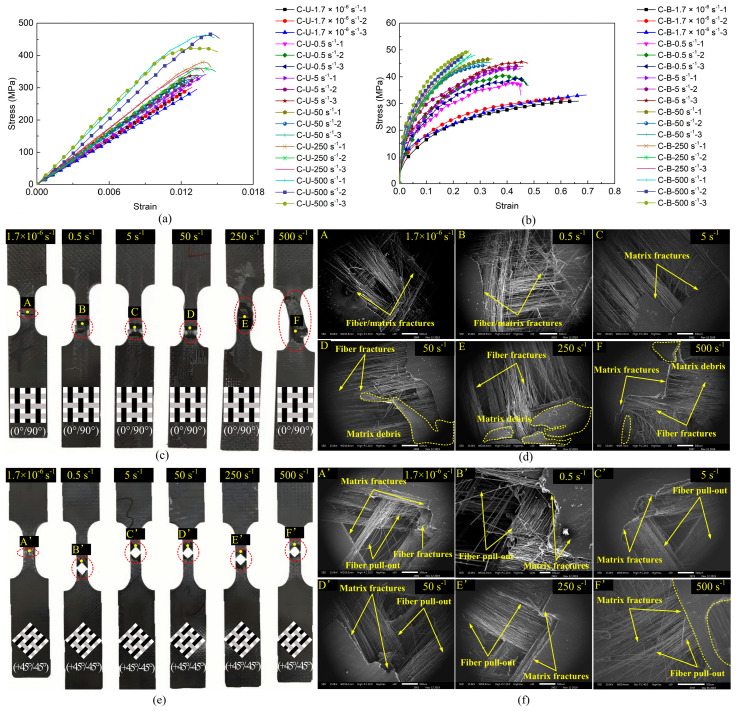
Mechanical responses and failure modes of CF/PP specimens: (**a**) uniaxial tensile stress–strain curves; (**b**) bias extension stress–strain curves; (**c**) macroscopic failure modes under uniaxial tension; (**d**) microscopic failure modes under uniaxial tension; (**e**) macroscopic failure modes under bias extension; (**f**) microscopic failure modes under bias extension.

**Figure 5:**
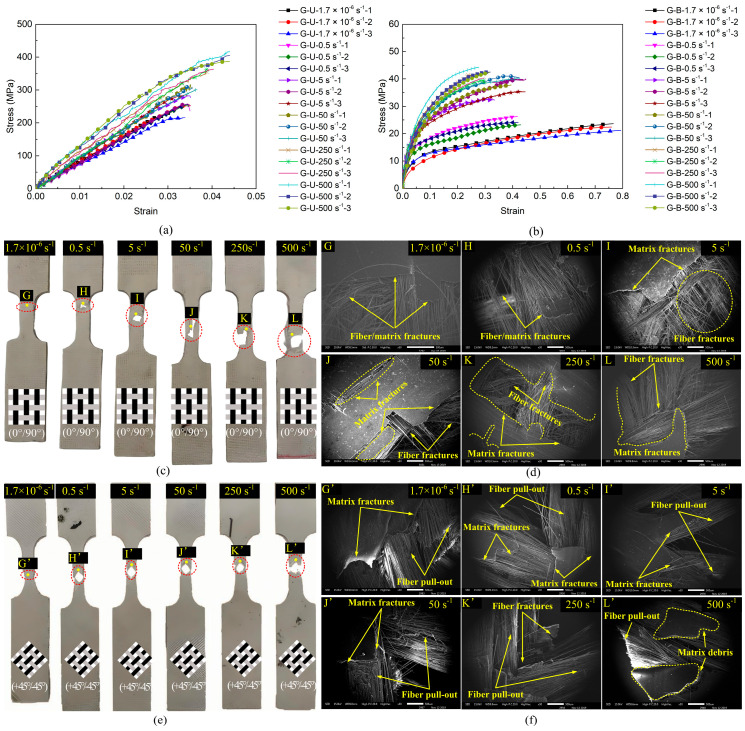
Mechanical responses and failure modes of GF/PP specimens: (**a**) uniaxial tensile stress–strain curves; (**b**) bias extension stress–strain curves; (**c**) macroscopic failure modes under uniaxial tension; (**d**) microscopic failure modes under uniaxial tension; (**e**) macroscopic failure modes under bias extension; (**f**) microscopic failure modes under bias extension.

Figure 4 and Figure 5 comparatively illustrate the mechanical responses, stress–strain curves, and failure modes of the CF/PP and GF/PP specimens across six strain rates (1.7 × 10^−6^ s^−1^~500 s^−1^), respectively. Table A1 and Table A2 summarize the modulus, strength, and failure strain parameters of all the CF/PP and GF/PP specimens (see Appendix A). In uniaxial tension testing of the CF/PP and GF/PP specimens, the stress–strain responses across six distinct strain rates exhibited linear elastic behaviors. The failure strain of the CF/PP specimens remained relatively constant at all strain rates. In contrast, the GF/PP specimens exhibited a marked increase in failure strain when the strain rate exceeded 250 s^−1^, as illustrated in Figure 4a and Figure 5a. During bias extension testing, the shear stress–strain curves for both the CF/PP and the GF/PP specimens revealed pronounced nonlinear deformation characteristics across six strain rates. Notably, the shear failure strain for both the CF/PP and the GF/PP specimens decreased as the strain rate increased, as depicted in Figure 4b and Figure 5b.

Figure 4c,d and Figure 5c,d show the macroscopic and microscopic failure morphologies of the CF/PP and GF/PP specimens subjected to the uniaxial tensile tests across six strain rates, respectively. It can be seen that the failure locations for all the CF/PP and GF/PP specimens were confined within the gauge length region, with failure modes near the fracture surfaces predominantly characterized by fiber breakage and matrix fragmentation. Numerous vertical fiber bundles were observed to be pulled apart accompanied by matrix cracking. At low strain rates, the fracture surfaces of both the CF/PP and the GF/PP specimens exhibited a relatively smooth morphology. However, the fracture surfaces exhibited a pronounced increase in roughness, which was simultaneously associated with an expansion of the failure zones as the strain rates increased.

Figure 4e,f and Figure 5e,f show the macroscopic and microscopic failure morphologies of the CF/PP and GF/PP specimens subjected to the bias extension tests across six strain rates, respectively. It can be found that the failure sites for both the CF/PP and the GF/PP specimens were centrally located within the gauge length region and exhibited a characteristic “V”-shaped fracture pattern. Owing to the 45° orientation between the fiber bundles and the loading direction, a substantial number of fiber bundles were extracted from the polypropylene matrix. This phenomenon resulted in extensive polypropylene fractures while limiting fiber fracture to minor occurrences. Fracture surfaces demonstrate progressively increased roughness in both resin matrix and fiber bundles with elevated strain rates.

### 3.2. Mechanical Parameters

**Figure 6:**
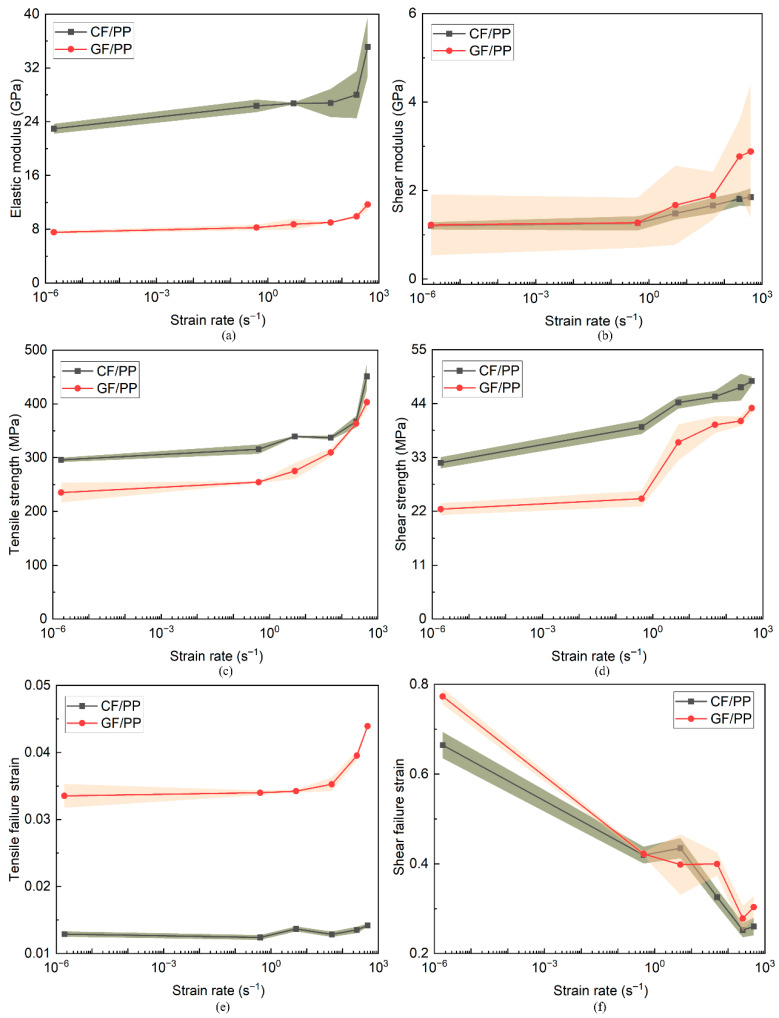
Comparisons in typical tensile and shear mechanical parameters of CF/PP and GF/PP specimens across six strain rates: (**a**) tensile elastic modulus; (**b**) shear modulus; (**c**) tensile strength; (**d**) shear strength; (**e**) tensile failure strain; (**f**) shear failure strains.

Based on the uniaxial tension and bias extension stress–strain curves of the CF/PP and GF/PP specimens (Figure 4a,b and Figure 5a,b), the elastic/shear modulus, tensile/shear strength, and tensile/shear failure strain parameters were derived and summarized in Table A1 and Table A2 (see Appendix A). Since each test condition was repeated three times, the average value of these replicates is reported as the ultimate mechanical parameter. The standard deviation (SD) calculated using Equation (A1) provided in Appendix A was used to quantify the variability of the experimental data. Figure 6 compares the difference in modulus, strength, and failure strain parameters between the CF/PP and GF/PP specimens across six strain rates, with error bands indicating the standard deviation. Figure 6a,d indicate that both the modulus and strength parameters of the CF/PP and GF/PP specimens demonstrated a progressive increase corresponding to the rise in strain rates. Specifically, the tensile/shear modulus and tensile/shear strength of the CF/PP specimens at a strain rate of 500 s^−1^ increased by 53.08%/53.6% and 52.5%/52.4% compared to those measured at 1.7 × 10^−6^ s^−1^, respectively. Similarly, the tensile/shear modulus and tensile/shear strength of the GF/PP specimens at a strain rate of 500 s^−1^ increased by 54.6%/113.4% and 71.5%/92.3% compared to those measured at a strain rate of 1.7 × 10^−6^ s^−1^, respectively. It is important to highlight that the modulus and strength values of CF/PP consistently exceed those of GF/PP under identical strain rate conditions. This difference is attributable to the superior stiffness and strength characteristics of carbon fibers.

However, the tensile failure strains of GF/PP are always larger than those of CF/PP owing to the superior toughness of glass fibers, as shown in Figure 6e. In contrast, the disparities in shear failure strains between the CF/PP and GF/PP specimens under the identical strain rate loading conditions are relatively small, and the shear failure strains of both the CF/PP and the GF/PP specimens exhibited a pronounced decline with increasing strain rates, as shown in Figure 6f. In particular, the shear failure strain of the CF/PP and GF/PP specimens at a strain rate of 500 s^−1^ decreased by 62.5% and 60.7%, respectively, relative to the values obtained at a strain rate of 1.7 × 10^−6^ s^−1^. This phenomenon can be attributed to the fact that both the CF/PP and the GF/PP specimens predominantly experienced fiber bundle shear deformations and polypropylene matrix plastic deformations. Given the comparable shear stiffness of carbon fibers and glass fibers (see Figure 6b), the majority of fiber bundles were extracted from the polypropylene matrix prior to experiencing adequate shear deformations. Consequently, the increase in strain rate induced a transition in the polypropylene from ductile to brittle behaviors and substantially reducing the shear failure strain.

### 3.3. Strain Rate Effects

The dynamic increase factor (DIF) is introduced to quantitatively establish a functional relationship between dynamic and quasi-static mechanical parameters, which can be determined as follows [42,43]:

(2) $$DIF=\left\{\left[tanh\left(\left(log\left(\frac{\dot{\varepsilon }}{{\dot{\varepsilon }}_{0}}\right)-A\right)\times B\right)\right]\times \left[\frac{C}{\left(C+1\right)/2}\right]+1\right\}\times \frac{C+1}{2}$$

where $\dot{\varepsilon }$ and ${\dot{\varepsilon }}_{0}$ denote the actual and the reference strain rate, respectively; A, B, and C are empirical parameters derived by fitting the experimental DIF data.

The correlation between the elastic and strength parameters at a given strain rate and those under quasi-static conditions can be formulated as follows [42,43]:

(3) $$E_{U_{\dot{\varepsilon }}}^{M}=E_{U_{{\dot{\varepsilon }}_{0}}}^{M}\times {DIF}_{UE}^{M}$$

(4) $$E_{B_{\dot{\varepsilon }}}^{M}=E_{B_{{\dot{\varepsilon }}_{0}}}^{M}\times {DIF}_{BE}^{M}$$

(5) $$S_{U_{\dot{\varepsilon }}}^{M}=S_{U_{{\dot{\varepsilon }}_{0}}}^{M}\times {DIF}_{US}^{M}$$

(6) $$S_{B_{\dot{\varepsilon }}}^{M}=S_{B_{{\dot{\varepsilon }}_{0}}}^{M}\times {DIF}_{BS}^{M}$$

where $E_{U_{\dot{\varepsilon }}}^{M}$, $S_{U_{\dot{\varepsilon }}}^{M}$, $E_{U_{{\dot{\varepsilon }}_{0}}}^{M}$, and $S_{U_{{\dot{\varepsilon }}_{0}}}^{M}$ denote the dynamic elastic modulus, dynamic tensile strength, quasi-static elastic modulus, and tensile strength under the uniaxial tension loading condition; while $E_{B_{\dot{\varepsilon }}}^{M}$, $S_{B_{\dot{\varepsilon }}}^{M}$, $E_{B_{{\dot{\varepsilon }}_{0}}}^{M}$, and $S_{B_{{\dot{\varepsilon }}_{0}}}^{M}$ denote the dynamic shear modulus, dynamic shear strength, quasit-static shear modulus, and shear strength under the bias extension loading condition.

**Figure 7:**
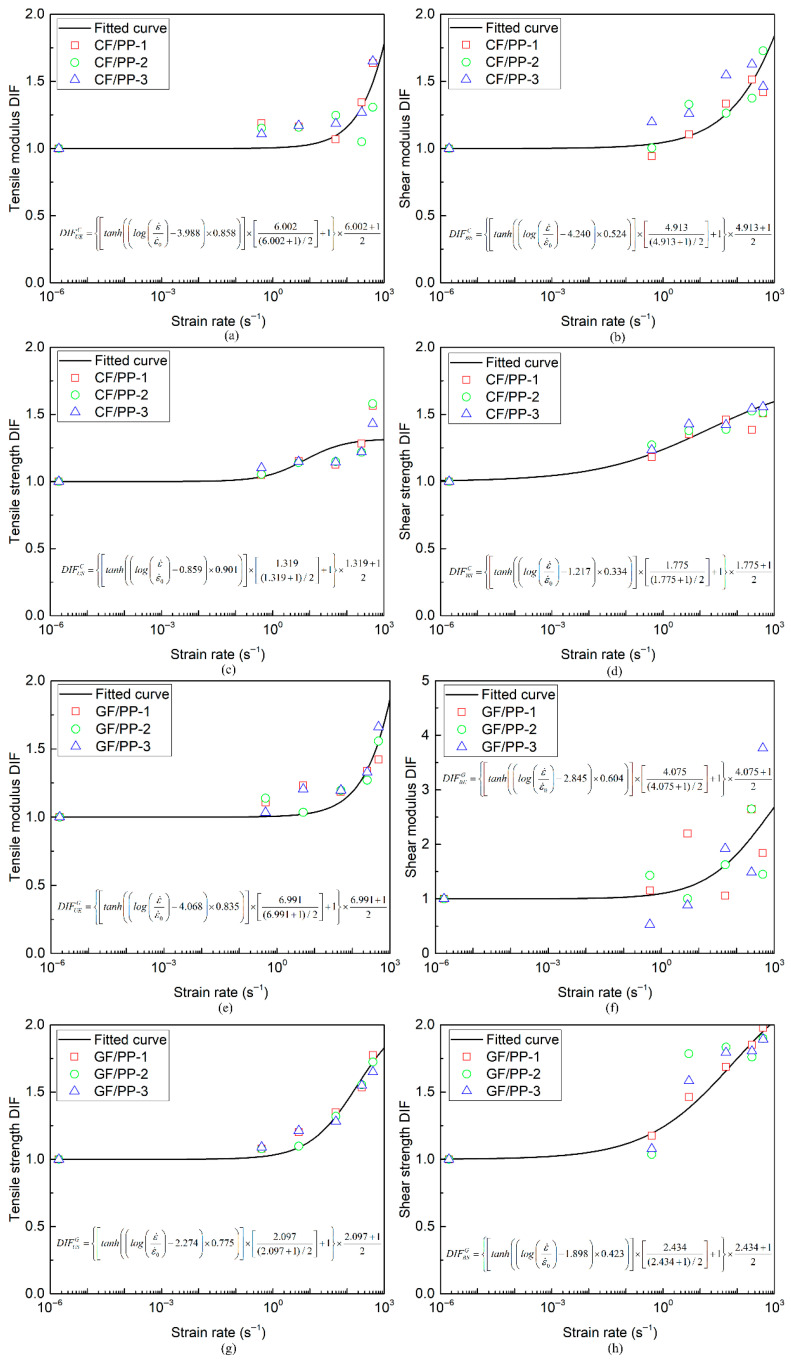
DIF value–strain rate curves of CF/PP and GF/PP specimens: (**a**) tensile modulus DIF of CF/PP; (**b**) shear modulus DIF of CF/PP; (**c**) tensile strength DIF of CF/PP; (**d**) shear strength DIF of CF/PP; (**e**) tensile modulus DIF of GF/PP; (**f**) shear modulus DIF of GF/PP; (**g**) tensile strength DIF of GF/PP; (**h**) shear strength DIF of GF/PP.

Utilizing the modulus and strength mechanical parameters of the CF/PP and GF/PP specimens obtained from the uniaxial tension and bias extension tests under six distinct strain rate conditions (refer to Table A1 and Table A2), the DIF values for the modulus and strength parameters of the CF/PP and GF/PP specimens were determined by employing Equations (3)–(6), as illustrated in Figure 7. It can be found when the strain rate exceeds 50 s^−1^, both the elastic and strength properties of the CF/PP and GF/PP specimens exhibit an upward trend with increasing strain rate, indicating that the mechanical behavior of polypropylene-based composites is sensitive to strain rate variations. Finally, the curve fitting of the DIF values was conducted based on Equation (2), yielding the corresponding parameters A, B, and C, as summarized in Table 3. The aforementioned parameters can offer substantial foundational support for developing rate-dependent constitutive models for CF/PP and GF/PP thermoplastic composites.

### 3.4. Discussion

It is worth noting that fiber pull-out is identified as one of the dominant failure mechanisms for both CF/PP and GF/PP laminates under dynamic shear loading conditions. This phenomenon is attributed to the weakened fiber/matrix interfacial bonding when subjected to high strain rates, which will ultimately compromise the integrity and energy absorption capacity of composite structures. To address this issue, several effective strategies can be implemented in further studies. Interfacial modification such as incorporating compatibilizers (for example, maleic anhydride grafted polypropylene), applying plasma treatment, or using silane coupling agents can substantially enhance fiber matrix adhesion and reduce pull-out. Moreover, structural optimization through the adoption of three-dimensional woven architectures, hybrid reinforcement layouts, or through thickness stitching can also effectively restrict fiber bundle sliding. Furthermore, processing improvements including elevated molding pressure, extended compressing time, or the introduction of nanofillers and elastomeric modifiers can promote better resin infiltration and strengthen the interface. These approaches may provide viable pathways for preventing fibers from being pulled out of the resin matrix, improving the dynamic shear performance and damage tolerance of thermoplastic composites.

The outcomes of this work not only enrich the fundamental understanding of strain rate effects in CF/PP and GF/PP thermoplastic composites but also provide direct guidance for engineering applications. In particular, the observed improvements in modulus and strength under dynamic conditions are highly relevant to the design of crashworthy automotive structures, such as bumper beams, B-pillars, and energy-absorbing components, where both light weight and safety must be balanced. Furthermore, the developed strain rate-dependent dynamic increase factor (DIF) offers reliable input parameters for finite element simulations and rate-dependent constitutive modeling, thereby accelerating the virtual design cycle and reducing experimental costs. Beyond the automotive sector, the recyclability and cost-effectiveness of PP-based composites support their sustainable application in lightweight transportation and other structural fields.

## 4. Conclusions

This study focused on the influences of strain rates on the mechanical responses of CF/PP and GF/PP thermoplastic composites. First, the thermal properties of polypropylene were characterized by the DSC and TGA tests. Next, the CF/PP and GF/PP laminate specimens were manufactured through the thermo-compression molding technology. Then, these specimens were subjected to the uniaxial tension and bias extension tests across six distinct strain rates (1.7 × 10^−6^ s^−1^, 0.5 s^−1^, 5 s^−1^, 50 s^−1^, 250 s^−1^, and 500 s^−1^). The influences of the strain rates on their macroscopic and microscopic failure modes, stress–strain curves, and elastic and strength mechanical parameters were analyzed. Finally, the functional relationship between dynamic and quasi-static mechanical parameters under different strain rates was quantitatively characterized by introducing the dynamic increase factor (DIF). The main conclusions can be drawn as follows:

The differential scanning calorimetry (DSC) analysis results revealed that the polypropylene matrix exhibited a melting temperature of approximately 166 °C. The thermogravimetric analysis (TGA) results demonstrated that the onset of thermal decomposition for the polypropylene matrix occurred near 400 °C, and the decomposition temperature ranged between 450 °C and 480 °C.

Under uniaxial tension tests, all the CF/PP and GF/PP specimens exhibited linear elastic behaviors across six distinct strain rates. The failure modes near the fracture surfaces were predominantly characterized by fiber breakages and matrix fractures. In contrast, under bias extension tests, all the CF/PP and GF/PP specimens exhibited pronounced nonlinear behaviors across six distinct strain rates. The failure modes are primarily characterized by fiber pull-out and matrix fractures.

For all the CF/PP and GF/PP specimens, both modulus and strength parameters demonstrated a progressive increase corresponding to the rise in strain rates. At 500 s^−1^, CF/PP exhibited 53.08%/53.6% and 52.5%/52.4% increases in tensile/shear modulus and tensile/shear strength versus 1.7 × 10^−6^ s^−1^, while GF/PP showed 54.6%/113.4% and 71.5%/92.3% improvements, respectively.

The dynamic increase factor (DIF) is introduced to quantitatively characterize the influence of loading rates on the modulus and strength properties. The critical parameters A, B, and C involved in the *DIF* formulation are precisely determined, which offers foundational support for developing rate-dependent constitutive models of CF/PP and GF/PP composites in future research.

## Figures and Tables

**Table 1:** Constituents and dimension parameters of the CF/PP and GF/PP prepregs.

Fiber	Matrix	Fiber Diameter	Yarn Width	Thickness	Density
Carbon	polypropylene	0.007 mm	0.9 mm	0.3 mm	1.08 g/cm^3^
Glass	polypropylene	0.01 mm	0.7 mm	0.3 mm	1.22 g/cm^3^

**Table 2:** Fundamental information of all test specimens.

Specimen No.	Material	Stacking Sequence	Velocity (m/s)	Strain Rate (s^−1^)
C-U-1.7 × 10^−6^	CF/PP	(0°,90°)_6_	3.3 × 10^−5^	1.7 × 10^−6^
C-U-0.5	CF/PP	(0°, 90°)_6_	0.01	0.5
C-U-5	CF/PP	(0°, 90°)_6_	0.1	5
C-U-50	CF/PP	(0°, 90°)_6_	1.0	50
C-U-250	CF/PP	(0°, 90°)_6_	5.0	250
C-U-500	CF/PP	(0°, 90°)_6_	10.0	500
C-B-1.7 × 10^−6^	CF/PP	(+45°, −45°)_6_	3.3 × 10^−5^	1.7 × 10^−6^
C-B-0.5	CF/PP	(+45°, −45°)_6_	0.01	0.5
C-B-5	CF/PP	(+45°, −45°)_6_	0.1	5
C-B-50	CF/PP	(+45°, −45°)_6_	1.0	50
C-B-250	CF/PP	(+45°, −45°)_6_	5.0	250
C-B-500	CF/PP	(+45°, −45°)_6_	10.0	500
G-U-1.7 × 10^−6^	GF/PP	(0°, 90°)_6_	3.3 × 10^−5^	1.7 × 10^−6^
G-U-0.5	GF/PP	(0°, 90°)_6_	0.01	0.5
G-U-5	GF/PP	(0°, 90°)_6_	0.1	5
G-U-50	GF/PP	(0°, 90°)_6_	1.0	50
G-U-250	GF/PP	(0°, 90°)_6_	5.0	250
G-U-500	GF/PP	(0°, 90°)_6_	10.0	500
G-B-1.7 × 10^−6^	GF/PP	(+45°, −45°)_6_	3.3 × 10^−5^	1.7 × 10^−6^
G-B-0.5	GF/PP	(+45°, −45°)_6_	0.01	0.5
G-B-5	GF/PP	(+45°, −45°)_6_	0.1	5
G-B-50	GF/PP	(+45°, −45°)_6_	1.0	50
G-B-250	GF/PP	(+45°, −45°)_6_	5.0	250
G-B-500	GF/PP	(+45°, −45°)_6_	10.0	500

Note: The symbols “C” and “G” correspond to the CF/PP and GF/PP specimens, respectively, while “U” and “B” indicate the uniaxial tension and bias extension tests, respectively. For instance, the designation C-U-1.7 × 10^−6^ signifies that the CF/PP specimen is subjected to uniaxial tension at a strain rate of 1.7 × 10^−6^ s^−1^.

**Table 3:** DIF values of modulus and strength parameters for CF/PP and GF/PP specimens.

Materials	Mechanical Properties	Symbols	A	B	C
CF/PP	Elastic modulus	$E_{U_{\dot{\varepsilon }}}^{CF}$	3.988	0.858	6.002
	Shear modulus	$E_{B_{\dot{\varepsilon }}}^{CF}$	4.240	0.524	4.913
	Elastic strength	$S_{U_{\dot{\varepsilon }}}^{CF}$	0.859	0.901	1.319
	Shear strength	$S_{B_{\dot{\varepsilon }}}^{CF}$	1.217	0.334	1.775
GF/PP	Elastic modulus	$E_{U_{\dot{\varepsilon }}}^{GF}$	4.068	0.835	6.991
	Shear modulus	$E_{B_{\dot{\varepsilon }}}^{GF}$	2.845	0.604	4.075
	Elastic strength	$S_{U_{\dot{\varepsilon }}}^{GF}$	2.274	0.775	2.097
	Shear strength	$S_{B_{\dot{\varepsilon }}}^{GF}$	1.898	0.423	2.434

## Data Availability

The original contributions presented in the study are included in the article, further inquiries can be directed to the corresponding author.

## Appendix A

Table A1 and Table A2 present a comprehensive summary of the mechanical testing results for the CF/PP and GF/PP specimens subjected to uniaxial and bias tension across six strain rate loading conditions, respectively. These tables include measurements of uniaxial tensile modulus, strength, and failure strain, as well as shear modulus, strength, and failure strain. To improve the reliability and validity of the data, each strain rate condition was evaluated in three replicates. The standard deviation (SD) was introduced to quantify the variability of the experimental data and is defined as follows:

(A1) $$SD=\sqrt{\frac{1}{n-1}\sum_{i=1}^{n}{\left(x_{i}-\bar{x}\right)}^{2}}$$

where SD is the sample standard deviation; n denotes the sample size; $x_{i}$ represents the i-th sample value; $\bar{x}$ is the mean value of all samples.

**Table A1:** Uniaxial tension and bias extension test results of CF/PP specimens.

Specimen No.	Modulus ± SD (GPa)	Strength ± SD (MPa)	Failure Strain ± SD
C-U-1.7 × 10^−6^-1	23.440	293.000	0.013
C-U-1.7 × 10^−6^-2	23.310	300.717	0.013
C-U-1.7 × 10^−6^-3	22.110	294.005	0.013
Average	22.950 ± 0.733	295.908 ± 4.196	0.013 ± 4.0 × 10^−4^
C-U-0.5-1	27.260	309.735	0.013
C-U-0.5-2	26.410	311.693	0.012
C-U-0.5-3	25.402	325.718	0.013
Average	26.357 ± 0.931	315.716 ± 8.718	0.012 ± 3.6 × 10^−4^
C-U-5-1	26.673	340.651	0.014
C-U-5-2	26.591	337.566	0.014
C-U-5-3	26.879	339.551	0.013
Average	26.714 ± 0.150	339.256 ± 1.564	0.014 ± 3.8 × 10^−4^
C-U-50-1	24.514	332.936	0.013
C-U-50-2	28.633	340.000	0.013
C-U-50-3	27.198	337.943	0.013
Average	26.782 ± 2.092	336.960 ± 3.633	0.013 ± 4.0 × 10^−4^
C-U-250-1	30.815	379.389	0.014
C-U-250-2	24.093	360.340	0.014
C-U-250-3	29.125	360.360	0.013
Average	28.011 ± 3.501	366.696 ± 10.992	0.014 ± 4.7 × 10^−4^
C-U-500-1	37.530	462.739	0.014
C-U-500-2	30.001	467.465	0.015
C-U-500-3	37.878	423.132	0.014
Average	35.136 ± 4.452	451.112 ± 24.347	0.014 ± 2.6 × 10^−4^
C-B-1.7 × 10^−6^-1	1.141	30.850	0.665
C-B-1.7 × 10^−6^-2	1.166	31.713	0.635
C-B-1.7 × 10^−6^-3	1.302	33.147	0.694
Average	1.203 ± 0.085	31.903 ± 1.160	0.665 ± 0.029
C-B-0.5-1	1.134	37.696	0.426
C-B-0.5-2	1.207	40.590	0.398
C-B-0.5-3	1.439	39.393	0.434
Average	1.260 ± 0.161	39.227 ± 1.454	0.420 ± 0.019
C-B-5-1	1.330	43.113	0.434
C-B-5-2	1.598	43.966	0.413
C-B-5-3	1.515	45.544	0.458
Average	1.481 ± 0.139	44.208 ± 1.233	0.435 ± 0.023
C-B-50-1	1.606	46.643	0.345
C-B-50-2	1.519	44.259	0.325
C-B-50-3	1.860	45.351	0.308
Average	1.662 ± 0.176	45.418 ± 1.193	0.326 ± 0.019
C-B-250-1	1.820	44.191	0.235
C-B-250-2	1.654	48.639	0.266
C-B-250-3	1.958	49.222	0.256
Average	1.810 ± 0.155	47.351 ± 2.752	0.252 ± 0.016
C-B-500-1	1.706	48.019	0.281
C-B-500-2	2.077	48.182	0.241
C-B-500-3	1.758	49.642	0.259
Average	1.847 ± 0.201	48.614 ± 0.894	0.260 ± 0.020

**Table A2:** Uniaxial tension and bias extension test results of GF/PP specimens.

Specimen No.	Modulus ± SD (GPa)	Strength ± SD (MPa)	Failure Strain ± SD
G-U-1.7 × 10^−6^-1	7.276	236.752	0.032
G-U-1.7 × 10^−6^-2	7.688	252.457	0.035
G-U-1.7 × 10^−6^-3	7.682	215.761	0.034
Average	7.549 ± 0.234	234.990 ± 18.411	0.034 ± 1.8 × 10^−3^
G-U-0.5-1	8.370	253.382	0.034
G-U-0.5-2	8.590	253.378	0.034
G-U-0.5-3	7.805	256.261	0.034
Average	8.255 ± 0.402	254.340 ± 1.664	0.034 ± 3.5 × 10^−4^
G-U-5-1	9.306	282.198	0.034
G-U-5-2	7.815	257.986	0.034
G-U-5-3	9.095	285.008	0.034
Average	8.739 ± 0.806	275.064 ± 14.856	0.034 ± 2.1 × 10^−4^
G-U-50-1	8.945	317.076	0.035
G-U-50-2	9.030	310.141	0.034
G-U-50-3	9.035	301.062	0.036
Average	9.003 ± 0.049	309.426 ± 8.031	0.035 ± 1.0 × 10^−3^
G-U-250-1	10.090	360.417	0.039
G-U-250-2	9.590	365.097	0.039
G-U-250-3	10.025	363.902	0.040
Average	9.902 ± 0.272	363.139 ± 2.432	0.040 ± 6.8 × 10^−4^
G-U-500-1	10.721	416.767	0.044
G-U-500-2	11.753	405.105	0.044
G-U-500-3	12.540	387.413	0.044
Average	11.671 ± 0.913	403.095 ± 14.780	0.044 ± 5.8 × 10^−5^
G-B-1.7 × 10^−6^-1	0.486	23.626	0.766
G-B-1.7 × 10^−6^-2	1.842	22.402	0.760
G-B-1.7 × 10^−6^-3	1.351	21.199	0.793
Average	1.227 ± 0.686	22.409 ± 1.213	0.773 ± 0.018
G-B-0.5-1	1.416	26.327	0.418
G-B-0.5-2	1.753	23.219	0.428
G-B-0.5-3	0.649	24.143	0.420
Average	1.273 ± 0.686	24.563 ± 1.596	0.422 ± 0.005
G-B-5-1	2.697	32.765	0.322
G-B-5-2	1.230	39.987	0.447
G-B-5-3	1.081	35.458	0.426
Average	1.669 ± 0.686	36.070 ± 3.650	0.398 ± 0.067
G-B-50-1	1.293	37.773	0.395
G-B-50-2	1.994	41.078	0.376
G-B-50-3	2.355	40.172	0.427
Average	1.881 ± 0.540	39.674 ± 1.708	0.400 ± 0.026
G-B-250-1	3.243	41.445	0.245
G-B-250-2	3.248	39.426	0.292
G-B-250-3	1.821	40.406	0.297
Average	2.771 ± 0.540	40.426 ± 1.010	0.278 ± 0.029
G-B-500-1	2.258	44.315	0.276
G-B-500-2	1.777	42.552	0.319
G-B-500-3	4.613	42.393	0.316
Average	2.883 ± 0.540	43.086 ± 1.067	0.304 ± 0.024
